# Early Respiratory Management of Respiratory Distress Syndrome in Very Preterm Infants and Bronchopulmonary Dysplasia: A Case-Control Study

**DOI:** 10.1371/journal.pone.0000192

**Published:** 2007-02-07

**Authors:** Arjan B. te Pas, Enrico Lopriore, Marissa J. Engbers, Frans J. Walther

**Affiliations:** Division of Neonatology, Department of Pediatrics, Leiden University Medical Center, Leiden, The Netherlands; University of Sydney, Australia

## Abstract

**Background:**

In the period immediately after birth, preterm infants are highly susceptible to lung injury. Early nasal continuous positive airway pressure (ENCPAP) is an attempt to avoid intubation and may minimize lung injury. In contrast, ENCPAP can fail, and at that time surfactant rescue can be less effective.

**Objective:**

To compare the pulmonary clinical course and outcome of very preterm infants (gestational age 25–32 weeks) with respiratory distress syndrome (RDS) who started with ENCPAP and failed (ECF group), with a control group of infants matched for gestational age, who were directly intubated in the delivery room (DRI group). Primary outcome consisted of death during admission or bronchopulmonary dysplasia (BPD).

**Results:**

25 infants were included in the ECF group and 50 control infants matched for gestational age were included in the DRI group. Mean gestational age and birth weight in the ECF group were 29.7 weeks and 1,393 g and in the DRI group 29.1 weeks and 1,261 g (p = NS). The incidence of BPD was significantly lower in the ECF group than in the DRI group (4% vs. 35%; P<0.004; OR 12.6 (95% CI 1.6–101)). Neonatal mortality was similar in both groups (4%). The incidence of neonatal morbidities such as severe cerebral injury, patent ductus arteriosus, necrotizing enterocolitis and retinopathy of prematurity, was not significantly different between the two groups.

**Conclusion:**

A trial of ENCPAP at birth may reduce the incidence of BPD and does not seem to be detrimental in very preterm infants. Randomized controlled trials are needed to test whether early respiratory management of preterm infants with RDS plays an important role in the development of BPD.

## Introduction

Early respiratory management (ERM) specifies ventilatory support from birth until stabilization during the first days of life. ERM starts with resuscitation in the delivery room (DR) and is primarily based on adequate ventilation. However, the recently updated international guidelines on neonatal resuscitation do not provide specific recommendations on how to provide ERM in very preterm infants due to lack of data.[1] Various ERM strategies are currently used to compensate for inadequate respiratory drive, surfactant deficiency and/or disturbances in the reabsorption of lung liquid after birth. Early nasal continuous positive airway pressure (ENCPAP), started directly after birth, is a gentle and effective non-invasive attempt to support non-compliant lungs.[2]–[6] It has been shown in animal studies that ENCPAP minimizes lung injury after preterm birth.[7], [8] During the immediate postnatal period the preterm, surfactant-deficient lung is highly susceptible to tissue injury and an exaggerated, unchecked inflammatory reaction can be triggered with the first manual breaths during resuscitation.[9]–[13] Several cohort studies report less requirement of mechanical ventilation when ENCPAP is used.[14] Nonetheless, a considerable percentage of preterm infants with RDS fails ENCPAP and requires intubation and mechanical ventilation during the first days of life.[15], [16] During this time period a potential improvement of lung function by surfactant treatment may have been missed as surfactant-deficient preterm infants respond better to surfactant treatment when it is given early in the course of their disease.[17], [18]

The aim of this case-control study was to compare the pulmonary course and outcome of very preterm infants with RDS who failed ENCPAP (ECF group) and a control group of infants, matched for gestational age, who were intubated in the DR (DRI group).

## Methods

### Patients

All inborn very preterm infants with a gestational age≤32 weeks admitted to the neonatal intensive care unit of the Leiden University Medical Center from January 2003 until December 2005 were identified. Limits of viability in the Netherlands are set at 25 weeks' gestation. Clinical data were collected from the hospital records. All infants with RDS who were supported with ENCPAP but failed and were intubated and ventilated within 72 hours after birth (ENCPAP failure = ECF group) were selected for the study group. Each infant in the ECF group was matched for gestational age (±1 week of gestation) with the next two consecutively born infants born with RDS who were intubated in the delivery room (delivery room intubation = DRI group). Diagnosis of RDS was based on clinical symptoms and chest X-ray findings. The following perinatal data were collected: birth weight, gestational age, gender, race, singleton or twin, maternal pregnancy disease, preterm premature rupture of the membranes, signs of chorioamnionitis (defined as maternal fever with at least one of the following symptoms: leucocytosis, tenderness of the uterus, fetal tachycardia, foul-smelling amniotic fluid), mode of delivery, intra-uterine growth retardation, antenatal steroids (defined as any administration of steroids irrespective of completion of therapy), umbilical arterial pH, resuscitation in the DR (none, oxygen administration, positive pressure ventilation with mask and bag, intubation and mechanical ventilation), 5 min Apgar score. Postnatal data collection included: ENCPAP (defined as NCPAP support within 15 minutes after admission to the neonatal intensive care unit), intubation within 72 hours, time and cause of intubation, clinical signs of RDS and staging by chest X-ray, duration of mechanical ventilation (first period and total period), type of ventilation (conventional ventilation only, rescue high frequency oscillation), surfactant treatment, administration of postnatal steroids, and occurrence of air leaks.

Primary outcome consisted of death during admission or bronchopulmonary dysplasia (BPD) based on the NICHD definition.[19] Secondary outcome was based on the presence of cerebral pathology detected by cranial ultrasound, retinopathy of prematurity, patent ductus arteriosus, and necrotizing enterocolitis.

### Respiratory management

If respiration was insufficient in the delivery room, positive-pressure ventilation was started with a self-inflating bag and mask during 30 seconds. At first inflation, pressures up to 30–40 cmH_2_0 could be given, thereafter not more than 20 cmH_2_O was allowed. If signs of respiratory distress persisted, the decision to intubate and start mechanical ventilation or to start ENCPAP (within 15 minutes) was dependant on RDS severity and clinical judgement of the attending neonatologist. After arrival in the neonatal intensive care unit, ENCPAP was given with nasopharyngeal tube or Infant Flow (EME Tricomed, Brighton, UK). CPAP-levels were titrated from 4 to 8 cmH_2_0 according to the degree of respiratory distress, assessed by observing the infant's chest retraction, effort of breathing, chest radiograph appearance, and oxygen requirement (stabilize oxygenation until arterial PO_2_>50 mmHg, while pH>7.20 and arterial PCO_2_<60 mmHg). Caffeine or theophylline treatment was started as soon as possible after birth in neonates<30 weeks of gestation and only after apnea emerged in more mature infants.

Intubation and mechanical ventilation were initiated when infants exhibited arterial hemoglobin saturations<88% or arterial PO_2_≤50 mmHg while receiving FiO_2_≥0.40 (corresponding with an AaPO_2_ ratio<0.22) and/or arterial PCO_2_>60 mmHg, pH<7.20, or when more than 4 apnea episodes occurred in one hour or mask ventilation was needed >2 times per hour. Surfactant was given when on mechanical ventilation with a mean airway pressure x FiO2 ratio>2. Infants with RDS were extubated as soon as a FiO2<0.3 and a mean airway pressure<7 cmH2O was reached. Immediately after extubation NCPAP was started using an infant flow device or nasopharyngeal tube. NCPAP was discontinued when the neonate remained stable with a capillary PCO_2_<60 mmHg, oxygen saturation>92% without supplementary O_2_ and no NCPAP for more than four consecutive hours. No changes in respiratory management occurred in the study period.

### Data analysis

Data are presented as mean±SD or number (percentage) and were analyzed with SPSS software (SPSS for windows, version 12.0, 2005, Chicago, IL). Analyses compared infants who failed ENCPAP treatment and were intubated within 72 hours of age (ECF group) and matched infants who were intubated immediately in the DR (DRI group). The null hypothesis was that the ECF group did not have a better pulmonary clinical course and outcome than the DRI group. The baseline characteristics of the two groups were compared using Student's t test for parametric and the Mann-Whitney U test for non-parametric comparisons for continuous variables, and the χ^2^ test for categorical variables. Reported p-values are two-sided; the level of significance is <0.05.

## Results

### Early respiratory management

**Figure 1:**
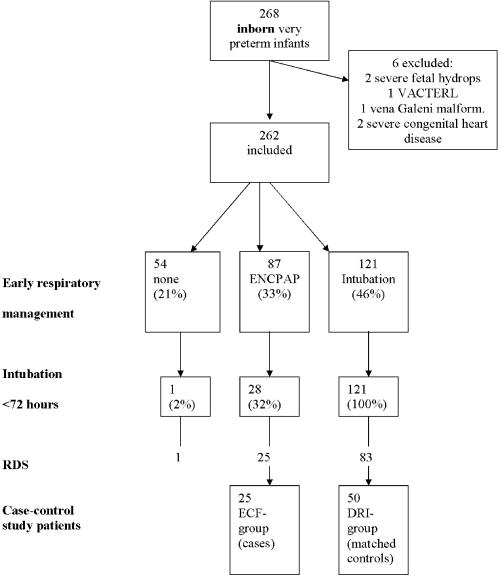
The distribution of need of ventilatory support in very preterm neonates (25–33 weeks of gestation). ENCPAP = early nasal continuous positive airway pressure; mask and bag: using a self-inflating bag.

**Figure 2:**
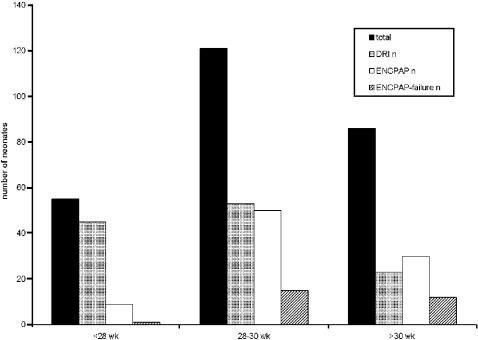
Distribution of delivery room intubation, ENCPAP and ENCPAP-failure plotted against gestational age. DRI = delivery room intubation; ENCPAP = early continuous positive airway pressure.

A total of 268 inborn very preterm infants (gestational age range between 25 and 32 weeks) were admitted consecutively to the neonatal intensive care unit between January 1, 2003 and December 31, 2005. Six infants were excluded because of severe congenital anomalies of the cardiac or respiratory system or anomalies incompatible with survival. The remaining 262 infants had a mean birth weight of 1318±385 g, mean gestational age of 29.3±1.9 weeks, and the mortality rate was 7% (19/262). The incidence of moderate or severe BPD [19] among survivors was 15% (37/243). ERM of the 262 included infants is shown in Figure 1. ENCPAP was started after arrival at the NICU in 83/87 (95%) infants and 4 (5%) of them received ENCPAP immediately in the DR. A nasopharyngeal tube was used 85% of the cases and an infant flow driver in 15%. There were no statistically significant differences in clinical failure between the two devices. The distribution of DRI, ENCPAP and ECF, plotted against gestational age is shown in Figure 2. Failure of CPAP occurred in 1/9 (11%) infants<28 weeks, in 15/50 (30%) infants of 28–30 weeks and in 12/30 (40%) infants>30 weeks of gestation. RDS was the principal cause of ECF (25/28 (89%) infants)

### ECF vs. DRI in very preterm neonates with RDS

RDS was diagnosed in 127/262 (48%) very preterm infants. In 42/127 (33%) infants with RDS, ENCPAP was started within 15 minutes after birth, and in 13 of them (31%) mask and bag ventilation was given in the delivery room. In 25/42 (59%) infants ENCPAP failed within 72 hours of age and they needed endotracheal intubation, ventilation and surfactant treatment (ECF-group) (mean time of intubation 14 hours after birth; range 0.3–65 hours). 83/127 (65%) infants with RDS were immediately intubated in the delivery room, of this group 50 infants formed the DRI-group according to described methods. The basic characteristics of the ECF-group and matched infants in the DRI-group were not significantly different (Table 1). Table 2 presents the pulmonary clinical parameters of the ECF and DRI groups. All patients with RDS intubated within 72 hours received surfactant, but significant more doses of surfactant were needed in the DRI-group.

**Table 1:** Comparison of characteristics of infants with RDS who failed treatment with ENCPAP (ECF) and matched infants who were intubated immediately after birth in the delivery room (DRI).

Characteristic	Group ECF *n = *25	Group DRI *n* = 50	Univariate analysis (*P)*
BW, g	1393±379	1261±395	NS
GA, wk	29.7±1.6	29.1±1.6	NS
Gender, male n (%)	9 (36)	27 (54)	NS
Umbilical arterial pH	7.28±0.13	7.22±0.13	NS
Prenatal steroids, n (%)	17 (68)	33 (66)	NS
Chorioamnionitis, n (%)	2 (8)	5 (10)	NS
PPROM, n (%)	6 (24)	15 (30)	NS
Toxaemia, n (%)	6 (24)	16 (32)	NS
Fetal distress, n (%)	9 (36 )	18 (36)	NS
IUGR, n (%)	4 (16)	11 (22)	NS
Caesarean section, n (%)	12 (48)	24 (48)	NS
Apgar, 5 min<7, n (%)	1 (4)	10 (20)	NS
Singletons, n (%)	13 (52)	20 (40)	NS

BW = birth weight; ENCPAP = early nasal continuous positive pressure; GA = gestational age; PPROM = preterm premature rupture of the membranes; IUGR = intra- uterine growth retardation.

**Table 2:** Pulmonary characteristics of neonates with RDS who failed ENCPAP treatment (ECF) and matched infants who were intubated in the DR (DRI).

Pulmonary clinical course parameters	ECF n = 25	DRI n = 50	Univariate analysis (*P)*
Surfactant, *n* doses	1.0±0.5	1.6±0.8	<0.0005
HFOV rescue, n (%)	3 (12)	15 (30)	NS
Total period of mechanical ventilation, days	3.9±5.6	7.0±9	NS
Pneumothorax, n (%)	1 (4)	3 (6)	NS
Dexamethasone, n (%)	1 (4)	7 (15)	NS

ENCPAP = early nasal continuous positive pressure; RDS = respiratory distress syndrome; HFOV = high frequency oscillation ventilation.

Primary outcome is shown in Table 3. Survivors in the DRI group had significantly more often moderate to severe BPD at 36 weeks of gestation than survivors in the ECF group. Mortality was 4% in both groups and all died due to respiratory failure.

**Table 3:** Primary outcome of infants who failed ENCPAP treatment (ECF) and matched infants who were intubated in the delivery room (DRI).BPD_mod-sev_ = moderate and severe bronchopulmonary dysplasia.

Primary outcome	ECF n = 25	DRI n = 50	Univariate analysis (*P)*
BPD mild	7	11	NS
BPD moderate	1	14	0.01
BPD severe	0	3	NS
BPD_mod-sev_, n (%)	1 (4)	17 (35)	0.004 OR 12.6 (95% CI 1.6–101)
Mortality, n (%)	1 (4)	2 (4)	NS

Comparison of secondary outcome between the ECF and DRI groups did not show significant differences in severe intraventicular hemorrhage (>grade 2) (3/24 (12.5%) vs. 3/49 (6.1%)), cystic periventricular leucomalacia (0/24 (0%) vs. 2/47 (4%)) and patent ductus arteriosus requiring medical or surgical closure (5/25 (20%) vs. 15/50 (30%)). Necrotizing enterocolitis requiring surgery and severe retinopathy of prematurity was absent in both groups.

## Discussion

ENCPAP proved to be a valuable initial ventilatory strategy as a significant percentage of very preterm infants (33%) was successfully managed with ENCPAP alone, even extremely preterm infants (<28 weeks). However, ENCPAP can also fail, requiring infants to be intubated within 72 hours after birth, mostly because of RDS. This study shows that infants in the ENCPAP failure group need less doses of surfactant and have a lower incidence of BPD compared to the matched DRI-infants. These findings are in agreement with animal studies, which have demonstrated that ventilatory management during the vulnerable period directly after birth may play an important role in the development of BPD. [7], [9]–[13], [20] A trial of ENCPAP at birth may be a good alternative for intubation in case of RDS and may reduce the incidence of BPD. Because the ECF group does not have more adverse outcomes and complications than the DRI group, it seems reasonable to avoid or postpone intubation if possible, as long as early surfactant rescue is given if intubation becomes mandatory.

Initial and early respiratory management in preterm infants is a hot topic in neonatology. The IFDAS trial, presented and published in abstract form, gives a good representation of the main approaches.[14] Many clinicians advocate elective intubation and early administration of surfactant.[17], [21] Others opt for an alternative approach of ENCPAP and selective surfactant treatment. Various non-randomized retrospective studies, with or without historic controls, report lower or no differences in rates of BPD in centers that try to avoid intubation and ventilation of preterm infants.[21] However, the inclusion criteria, including gestational age of the preterm infants, the methods used to deliver NCPAP, and the levels of CPAP used vary between the studies. The interpretation of “early” NCPAP varies from starting in the delivery room directly after resuscitation to starting in the neonatal intensive care unit when signs of RDS appear.[21] ENCPAP and selective surfactant is an accepted alternative, but a Cochrane review of trials of prophylactic ( = early) NCPAP recently concluded that there is currently insufficient information to evaluate the effectiveness of prophylactic NCPAP in very preterm infants.[22] Retrospective studies in which ENCPAP is started in the delivery room have shown a reduction in incidence of BPD.[3], [4], [6] To the best of our knowledge, this is the first case-control study comparing ENCPAP-failure and DR intubation in case of RDS. Similar to our study, Aly *et al.* retrospectively compared outcome of ENCPAP-failure and delivery room intubation in very preterm infants. However, they did not report the incidence of RDS in the total group and found, after controlling for basic characteristics, no difference in outcome, except for more NEC in the ENCPAP-failure group.[16] We found no significant differences in secondary outcome, including NEC. Lindner *et al.* reported a significant decrease in DR intubation when a new ventilatory strategy was introduced, consisting of a continuous prolonged pressure-controlled inflation followed by CPAP at 4–6 cmH2O.[11] Their study was not designed to test the difference in outcome between direct intubation and ENCPAP-failure. Nevertheless, in case of RDS, the incidence of BPD was 40% when infants were intubated in the delivery room compared to 22% when intubated later.[6] In contrast to these reports and also to our findings, the infant flow driver and surfactant (IFDAS) trial found no reduction in BPD when ENCPAP was used. Lack of significance may have been the result of a lack of power due to randomisation of the 237 infants of 27–29 weeks of gestation to 4 treatment groups. [14]

The period directly after birth is a very vulnerable phase and unnecessary intubation and ventilation may damage the upper airway and cause iatrogenic respiratory distress.[13], [20]. The use of a self-inflating bag prior to intubation may also induce lung injury as PEEP can not be regulated and an accurate peak inspiratory pressure is difficult to achieve. [23], [24] Acute ventilator-induced lung injury sustained shortly after birth can trigger the inflammatory cascade, which may progress to BPD.[7]–[13] Animals studies have shown that this inflammatory process may occur with the first manual breaths during resuscitation and compromise the effect of subsequent surfactant rescue treatment.[9];[10];[12] Even administration of prophylactic surfactant does not protect against the adverse affects of large lung inflations at birth.[11] In addition, lung injury after birth can be minimized by using ENCPAP instead of intubation and mechanical ventilation.[7], [8]

Symptoms of respiratory distress at birth in preterm infants may solely be due to their efforts to clear fluid from their lungs. Their ability to do so can be limited by surfactant deficiency and their soft and flexible chest wall makes preterm infants less able to generate sufficiently high pressures to achieve effective lung expansion.[25], [26] Nevertheless, expectant management (without direct intubation) may show that some preterm neonates are able to manage this with NCPAP assistance only. Once the alveoli are aerated, breathing needs less effort and distress symptoms may diminish. Some preterm infants who are intubated and ventilated in the DR may have had respiratory distress appropriate to a transitory phase, which eventually led to pulmonary complications due to “iatrogenic” RDS.

Compared to other reports, we found a higher percentage of ENCPAP-failure (32%) in our study, mostly due to RDS (89%).[4], [15], [16], [27] This difference may be due to the lower threshold for intubation and surfactant used at our institution. Preterm infants with RDS at our neonatal intensive care unit are intubated and ventilated as soon as the FiO2 is>40% or PaCO2>8.0 kPa and a maximum ENCPAP (8 cmH2O) is reached. Treatment with surfactant is considered when mean airway pressure x FiO2>2. In other reports, the FiO2 threshold for intubation was higher and varied from ≥0.6 to 1.0.[4], [15], [16], [27] The threshold for intubation and surfactant treatment when RDS has been established is an important issue. Prophylactic or early surfactant treatment of infants requiring mechanical ventilation is more effective than late rescue treatment.[17], [18], [28] However, with prophylactic treatment a certain amount of (surfactant sufficient) preterm infants are over-treated. Conversely, late rescue (FiO2≥0.6) with surfactant in an infant who is quickly deteriorating because of RDS may be disadvantageous and deleterious.[29], [30] Another explanation for the higher incidence of ENCPAP-failure in this study is the starting point of ENCPAP. Even if the lung is opened in the DR, with or without the help of mask and bag ventilation, a noncompliant surfactant-deficient lung has a tendency to collapse and lung volume is not maintained if CPAP/PEEP is not given directly to keep the lung open. In most infants in our study, ENCPAP was only started after transportation to the neonatal intensive care unit, which is adjacent to the delivery room, allowing the lung to collapse.

We are aware that the retrospective nature of this case-control study forms an important limitation. A selection-bias may have occurred in the DR when the decision was made to intubate or not and infants in the DRI-group may represent the sickest neonates with more severe RDS and higher risk for BPD. Although we found no significant differences in pulmonary clinical course parameters between the ECF and DRI groups, lack of significance can be explained by the small sample size of the study.

In summary, this study suggests that a trial of ENCPAP at birth is not detrimental and may be justified in case of RDS, providing early surfactant rescue is given if the infant needs to be intubated and ventilated. Based on this study, randomized controlled trials are warranted to test the hypothesis that early respiratory management of preterm neonates with RDS plays an important role in the development of BPD. Recently published or started randomized trials of ENCPAP are few.[31], [32] In 2005 we started a randomized controlled trial (EFURCI-trial, Early Functional Residual Capacity Intervention, ISRCTN 12757724) to investigate in preterm infants≤32 weeks whether sustained inflation with a mechanical device (Neopuff®) and ENCPAP will be a more effective management strategy in very preterm neonates than conventional intervention and will reduce the requirement for mechanical ventilation and surfactant treatment.
